# Mitochondrial Genome of the Korean Endemic *Arabis takesimana* (Brassicaceae): Addition to the Genomic Resources and Evolutionary Insights

**DOI:** 10.3390/biology15191762

**Published:** 2026-10-02

**Authors:** Asif S. Tamboli, Jin-Suk Youn, Suhas K. Kadam, Manoj M. Lekhak, Jan Bocianowski

**Affiliations:** 1 Research Institute for Dok-do and Ulleung-do Island, Kyungpook National University, 80 Daehak-ro, Buk-gu, Daegu 41566, Republic of Korea; 2 Independent Researcher, Dighanchi, Sangli 415315, Maharashtra, India; 3 Invasive Alien Species Team, National Institute of Ecology, 1210 Geumgang-ro, Maseo-myeon, Seocheon-gun 33657, Republic of Korea; okita0522@naver.com; 4 Department of Agricultural Biotechnology, National Institute of Agricultural Sciences, Rural Development Administration, Jeonju 54874, Republic of Korea; suhas397@gmail.com; 5 Angiosperm Taxonomy Laboratory, Department of Botany, Shivaji University, Kolhapur 416004, Maharashtra, India; mml_botany@unishivaji.ac.in; 6 Department of Mathematical and Statistical Methods, Poznań University of Life Sciences, Wojska Polskiego 28, 60-637 Poznań, Poland

**Keywords:** *Arabis takesimana*, Brassicaceae, comparative mitogenomics, mitochondrial genome, phylogenomics

## Abstract

Brassicaceae is a major model system for studies of plant evolution, systematics, comparative genomics, and speciation; however, mitochondrial genomic resources remain limited compared to plastid genomic resources, constraining investigations of mitochondrial genome evolution. Here, we report the mitogenome of the Korean endemic species *Arabis takesimana*, representing only the third available mitogenome for the genus *Arabis*. The mitogenome is 291,633 bp in length with a GC content of 45.3%. It was assembled with an average sequencing depth of 298.9×. It contains 58 annotated genes, including 34 protein-coding genes, three rRNA genes, and 21 tRNA genes. A 5.15 kb mitochondrial plastid DNA insertion (MIPT) region contributes four intact plastid-derived tRNA genes (*trn*A, *trn*L-CAA, *trn*S-GGA, and *trn*N-GUU) and a partial *rrn18* gene. The mitogenome also contains 21.83 kb of transposable element (TE) sequences and 44.03 kb of repetitive DNA, representing 7.48% and 15.09% of the genome, respectively. Comparative analysis of 32 Brassicaceae mitogenomes revealed 30 conserved mitochondrial protein-coding genes shared across the sampled taxa, whereas *rpl2*, *rpl10*, *rps1*, *rps10*, *rps13*, *rps14*, *rps19*, *sdh3*, and *sdh4* exhibited lineage-specific patterns of truncation, pseudogenization, or loss. Linear regression revealed a strong positive association between mitogenome size and TE content (*R*^2^ = 0.7245), whereas MIPT content (*R*^2^ = 0.09146) and repetitive DNA (*R*^2^ = 0.1896) showed weaker associations with mitogenome size. Nucleotide diversity analyses identified highly variable mitochondrial loci with potential as molecular markers. Phylogenetic analyses based on conserved mitochondrial protein-coding genes resolved sampled Brassicaceae species with strong support and established the phylogenetic placement of *A. takesimana*, highlighting the utility of mitogenomic data for evolutionary, phylogenetic, and comparative genomic studies within the family.

## Simple Summary

Plant mitochondrial genomes (mitogenome) provide valuable information for understanding plant evolution, diversity, and relationships, but they remain less well studied than plastid genomes. In this study, we generated the mitogenome of *Arabis takesimana*, a Korean endemic species and only the third *Arabis* species with an available mitogenome. The mitogenome is about 292 kb long and contains genes essential for mitochondrial function, along with plastid-derived DNA and repetitive sequences. Linear regression revealed a strong association between transposable-element content and mitogenome size across the sampled Brassicaceae species, whereas plastid-derived DNA and repetitive sequences showed weaker associations. We also identified variable mitochondrial genes that may be useful as molecular markers and found evidence of different evolutionary pressures acting on mitochondrial genes. Our study provides a valuable new genomic resource for *A. takesimana* and offers initial insights into mitochondrial genome evolution within the sampled Brassicaceae lineages. Because taxonomic sampling was limited, these findings should be considered preliminary. Nevertheless, the study provides useful information for future research on plant mitochondrial evolution, phylogenetics, and comparative genomics.

## 1. Introduction

Brassicaceae is one of the largest and most economically and scientifically important families of flowering plants, comprising approximately 4158 species distributed among more than 357 genera and including numerous crops as well as the model plant *Arabidopsis thaliana* (L.) Heynh. [1,2]. Because of its extensive morphological diversity, repeated polyploidization, and well-resolved taxonomy, Brassicaceae has become a premier model system for investigating plant evolution, systematics, comparative genomics, and speciation [1,2]. Despite considerable advances in nuclear and plastid phylogenomics of Brassicaceae, mitochondrial genomic resources remain comparatively scarce, limiting comprehensive investigations of mitochondrial genome evolution across the family [1]. Plant mitochondrial genome exhibits remarkable structural complexity characterized by extensive genome rearrangements, repeat-mediated recombination, intracellular DNA transfer, variable genome sizes, foreign DNA acquisition, and frequent gain or loss of genes, making them valuable systems for studying organelle genome evolution and cytonuclear interactions [3,4,5]. Furthermore, mitogenomes are valuable resources for investigating cytoplasmic male sterility, molecular evolution, phylogenetic inference, and adaptive evolution, all of which have important implications for crop improvement and evolutionary biology [5,6].

To date, complete mitogenome assemblies are available for only a limited number of Brassicaceae species, leaving many genera poorly represented and restricting comparative analyses of genome organization, repeat dynamics, sequence transfer, RNA editing, and structural evolution [6,7,8,9,10,11,12,13,14,15]. Therefore, generating additional mitogenome resources from phylogenetically diverse Brassicaceae species is essential for improving our understanding of mitogenome evolution, resolving evolutionary relationships using independent organellar evidence, elucidating cytonuclear genome evolution, and establishing a comprehensive genomic framework for future evolutionary, systematic, and breeding studies within the family.

*Arabis* L. (Brassicaceae) is a temperate Northern Hemisphere genus whose circumscription has undergone substantial revision following molecular phylogenetic studies [16,17,18]. Although the genus was historically estimated to comprise 100–180 species [16,17], phylogenetic analyses demonstrated that *Arabis* was polyphyletic, resulting in the transfer of numerous species to other genera within Brassicaceae [19,20,21,22,23,24,25]. Consequently, the genus is now recognized to include approximately 60–70 species [24,26,27].

In Korea, the taxonomy of *Arabis* has also been revised extensively. From the 12 taxa originally reported by Nakai [28], subsequent taxonomic revisions and generic reassignments reduced the number of recognized species. The description of *A. erecta* Y.Y. Kim & C.G. Jang by Kim et al. [29] resulted in the current recognition of six species: *A. columnalis* Nakai, *A. erecta*, *A. hirsuta* (L.) Scop., *A. serrata* Franch. & Sav., *A. stelleri* DC., and *A. takesimana* Nakai, Bot. Mag. (Tokyo) 32:244 (1918). *A. takesimana* is a perennial herb endemic to Ulleungdo Island, Republic of Korea, where it is considered one of the characteristic endemic members of the island flora [28,30]. More recently, complete chloroplast genome sequencing demonstrated that *A. takesimana* possesses a highly conserved plastome structure and gene content typical of Brassicaceae and confirmed its sister relationship with *A. hirsuta*, *A. flagellosa* Miq., *A. nipponica* (Franch. & Sav.) H. Boissieu, and *A. paniculata* Franch. and *A. stelleri* in plastome-based phylogenetic analyses [31]. Despite these advances, genomic studies of *A. takesimana* have been largely restricted to the plastid genome, and until the present study, no complete mitogenome had been reported, limiting investigations of mitogenome evolution.

The present study aimed to assemble and characterize the mitogenome of *A. takesimana* and to place it in a broader comparative framework within Brassicaceae. Although plant mitochondrial genomes contain highly conserved essential genes, they exhibit considerable variation in genome size, structure, gene content, and repetitive and foreign DNA sequences. Comparative analysis across taxonomically diverse Brassicaceae lineages can therefore provide insights into the patterns and processes underlying mitochondrial genome evolution and diversification. To achieve broad comparative coverage, we analyzed *A. takesimana* together with 31 publicly available Brassicaceae mitogenomes, encompassing mitochondrial genomic diversity across multiple lineages within the family. We compared these mitogenomes to assess variation in genome size, gene content, structural features, mitochondrial plastid-derived sequences (MIPTs), transposable elements (TEs), repetitive DNA, and single-copy regions. We further investigated gene loss and pseudogenization to identify lineage-specific patterns of genome evolution and analyzed nucleotide diversity and selective pressures in mitochondrial protein-coding genes to examine patterns of sequence conservation and diversification. Finally, we evaluated the phylogenetic utility of mitochondrial coding sequences for resolving relationships among Brassicaceae lineages. By integrating genome structure, gene content, repetitive and foreign DNA, sequence evolution, and phylogenetic information across multiple Brassicaceae lineages, this study provides a broader perspective on mitochondrial genome diversity and evolution within the sampled taxa. Given the limited taxonomic sampling, these findings remain preliminary and provide a comparative framework for future studies incorporating broader taxonomic sampling and additional genomic data to further investigate mitochondrial genome evolution and phylogenetic relationships in Brassicaceae.

## 2. Materials and Methods

### 2.1. Mitogenome Assembly and Annotation

Raw Illumina 151 bp paired-end reads of *A. takesimana* from Tamboli et al. [31] were used for mitogenome assembly. Total Raw Reads (42,327,400) were first mapped against the plastome of *A. takesimana* (GenBank accession no. OR813051) to obtain plastome-filtered reads. The filtered reads were initially assembled using NOVOPlasty v4.3.1 [32] with a k-mer size of 23, using the *cox*1 gene from *A. alpina* as the seed and its mitochondrial genome (NC_037070) as a reference. Although NOVOPlasty performs *de novo* assembly, the closely related reference genome was used to guide assembly, particularly for resolving duplicated and repetitive regions. NOVOPlasty assembly produced seven contigs. The resulting contigs were subjected to BLAST (https://blast.ncbi.nlm.nih.gov/Blast.cgi, accessed on 21 May 2026) searches against the mitogenomes of *A. alpina* (NC_037070) and *A. hirsuta* (OZ053314), confirming all seven contigs as putative mitochondrial sequences. The plastome-filtered reads (38,476,074) were subsequently mapped to these seven mitochondrial contigs using Bowtie2 v2.5.5 [33], and the mapped reads (584,667) were assembled with GetOrganelle v1.7.7.1 [34] using parameters-R 30-k 21,55,85,115,127-F embplant_mt. All software (Bowtie2, NOVOPlasty, and GetOrganelle) was accessed via the Galaxy Australia portal (https://usegalaxy.org.au/, accessed on 21 May 2026) [35]. The resulting circularized mitochondrial genome represents an assembly model supported by the available sequencing data. The mitogenome was validated by remapping the plastome-filtered reads to the assembled genome and confirming the presence of all core mitochondrial protein-coding genes (CDSs). Genome annotation was performed with PMGA [36] and manually curated in Geneious Prime 2026.0.1 (https://www.geneious.com/) [37]. Finalized annotated mitogenome was submitted to GenBank, and accession numbers were obtained. Circular genome map were generated using OGDRAW v1.2 [38].

### 2.2. Taxon Sampling for Comparative Mitogenomics

To compare the mitogenome of *A. takesimana* with those of other *Arabis* species and available representatives of the Brassicaceae, a total of 32 mitogenomes were included in the comparative analysis. The dataset comprised three *Arabis* species, 29 additional Brassicaceae taxa. Prior to comparative analyses, all mitogenomes were re-annotated using the PMGA tool (http://www.1kmpg.cn/pmga/) [36], followed by manual curation and refinement in Geneious Prime 2026.0.1 to ensure annotation consistency and accuracy. A complete list of the analyzed species and their corresponding mitogenome GenBank accession numbers is provided in the Supplementary Material (Table S1).

### 2.3. Comparative Analysis of Mitogenome Gene Content

The CDSs, together with the numbers of rRNA and tRNA genes, were analyzed across all 32 Brassicaceae mitogenomes, including that of *A. takesimana*. Each coding sequence (CDS) was evaluated and classified as intact, truncated, pseudogenized, or missing. An intact CDS was defined as a complete coding sequence with an uninterrupted open reading frame. A truncated CDS was defined as a shortened coding sequence retaining at least 50% of the length of its corresponding intact homolog, typically resulting from a premature stop codon near the original start codon, the use of an alternative downstream start codon, or the presence of an additional premature stop codon within the coding region. A CDS was classified as pseudogenized if it was either severely shortened or had lost its original reading frame due to frameshift mutations or multiple disruptive mutations, rendering it nonfunctional. A gene was considered missing when no recognizable gene fragment was detected or when only residual fragments shorter than 100 bp were present.

### 2.4. Analysis of Structural Traits of Sampled Brassicaceae Mitogenomes

Putative transposable elements (TEs) in each mitogenome was identified using the CENSOR web server (https://www.girinst.org/censor/index.php, accessed on 20 June 2026) [39] with default parameters, using the *Viridiplantae* reference sequence library as described by Park et al. [40]. Mitochondrial DNA segments of plastid origin (MIPTs) were identified and quantified using BLAST+ v2.17.0 with default settings by aligning each mitogenome against its corresponding plastome (Table S1). For each mitogenome, both the number and total length of MIPTs were recorded, and only plastid-derived fragments longer than 100 bp were included in the analysis. The repetitive regions of each mitogenome were characterized by analyzing the distribution of two classes of repeats: tandem and dispersed repeats. Tandem repeats were identified using Tandem Repeats Finder v4.09 [41] with default parameters. Dispersed repeats were detected using BLASTN by performing self-comparisons of each mitogenome, with a word size of 7 and an *E*-value threshold of 1 × 10^−6^, following the approach described by Guo et al. [42]. This strategy enabled the detection of dispersed repeats as short as 30 bp, and all BLAST hits were retained for subsequent analysis. The coordinates of both tandem and dispersed repeats were annotated in Geneious Prime 2026.0.1. Overlapping repeat regions were excluded from the final calculations to avoid redundancy, and the proportions of repetitive and single-copy DNA were subsequently determined for each mitogenome.

### 2.5. Evaluation of Nucleotide Diversity and Selective Pressure

To assess nucleotide diversity among the 30 intact mitochondrial CDS (*atp1*, *atp4*, *atp6*, *atp8*, *atp9*, *ccmB*, *ccmC*, *ccmFc*, *ccmFN*, *cob*, *cox1*, *cox2*, *cox3*, *matR*, *mttB*, *nad1*, *nad2*, *nad3*, *nad4*, *nad4L*, *nad5*, *nad6*, *nad7*, *nad9*, *rpl5*, *rpl16*, *rps3*, *rps4*, *rps7*, and *rps12*) shared across 32 Brassicaceae species, the CDSs of each gene were extracted using Geneious Prime 2026.0.1. For nucleotide diversity analysis, each CDS was aligned individually using MAFFT v7 [43], and the resulting alignments were concatenated into a single dataset. Nucleotide diversity was then estimated using DnaSP v6.10 [44]. For selection pressure analysis, each CDS was realigned using the MUSCLE algorithm [45] implemented in MEGA X [46] with the align by codons option to preserve the correct reading frame. The ratio of the nonsynonymous substitution rate (Ka) to the synonymous substitution rate (Ks) (Ka/Ks) for each CDS was subsequently calculated using DnaSP v6.10 [44].

### 2.6. Phylogenetic Analysis

A total of 30 intact mitochondrial CDSs shared among 32 Brassicaceae species and one outgroup species from the Bataceae, *Batis maritima* were used for phylogenetic analyses. The CDS of each gene was extracted, aligned individually using MAFFT v7 [43], and subsequently concatenated into a single sequence matrix for phylogenetic reconstruction. Maximum likelihood (ML) analysis was performed using RAxML-HPC v8.0 [47] on the XSEDE platform through the CIPRES Science Gateway [48] under the GTR+G nucleotide substitution model. Branch support was assessed using the rapid bootstrap algorithm with 1000 bootstrap replicates.

Bayesian inference (BI) analysis was conducted using MrBayes v3.2.7a [49] via the CIPRES Science Gateway. Prior to the BI analysis, the best-fitting nucleotide substitution model for the concatenated dataset was selected based on the Akaike Information Criterion (AIC) using jModelTest 2 [50], which identified GTR+I+G as the optimal model. Two independent Markov chain Monte Carlo (MCMC) analyses were run for 1,000,000 generations, with trees sampled every 1000 generations. Convergence was considered to have been reached when the average standard deviation of split frequencies fell below 0.01. The first 25% of sampled trees were discarded as burn-in, and the remaining trees were used to construct the Bayesian consensus tree. Posterior probability values were calculated to assess branch support.

## 3. Results

### 3.1. Gene Content and Plastid-Derived Sequences in the A. takesimana Mitogenome

**Figure 1:**
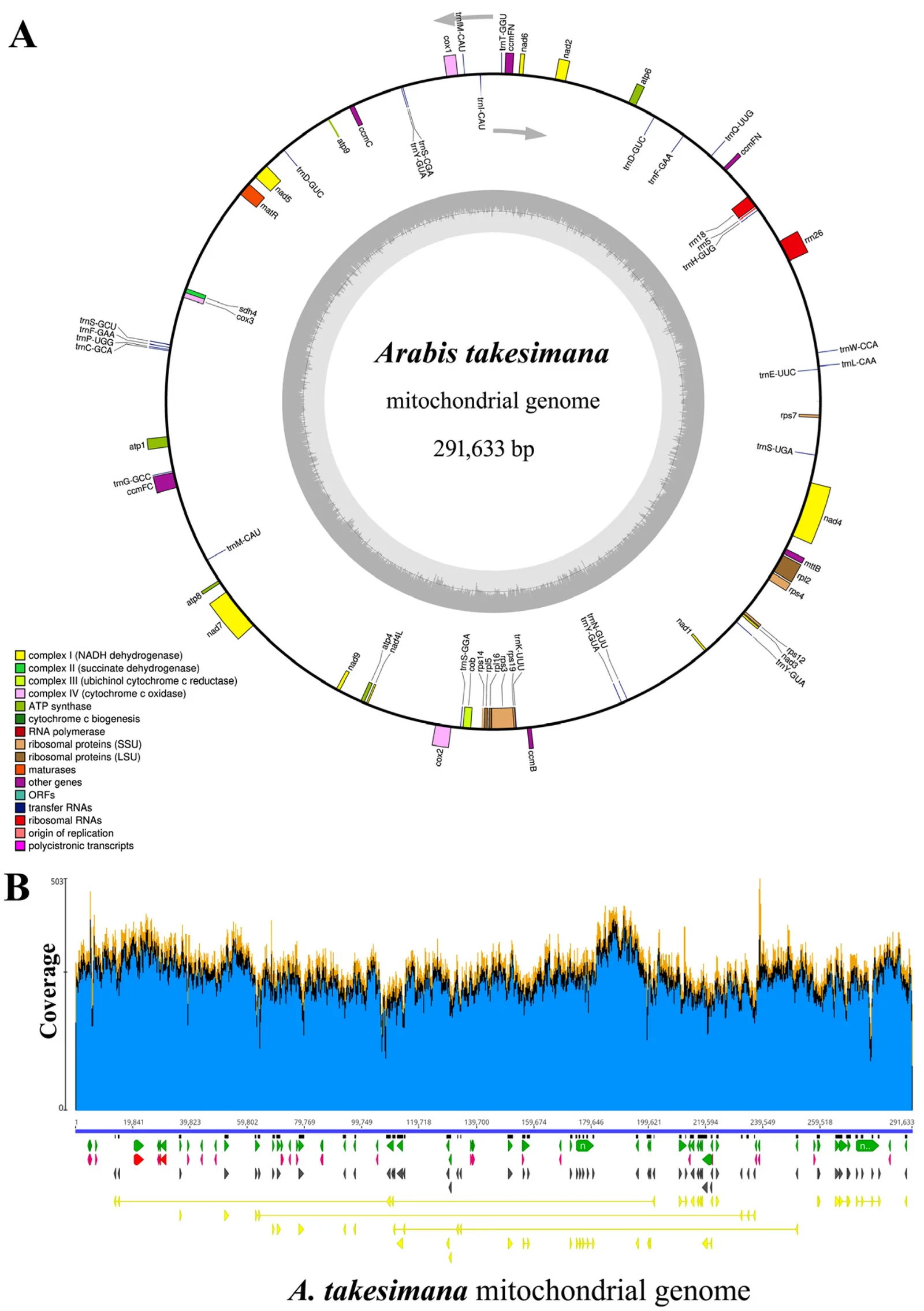
Circular map and sequencing coverage of *A. takesimana* mitogenome. (**A**) Circular map of the *A. takesimana* mitogenome. Genes located outside the circle are transcribed in the counterclockwise direction, whereas genes inside the circle are transcribed in the clockwise direction. Genes are color-coded according to their functional categories. The gray inner histogram represents GC content across the genome. (**B**) Coverage plots shows the depth of sequencing reads mapped back to the assembled mitogenome, with blue bars representing read depth and a black line indicating average coverage. The uniformity of coverage supports the completeness and accuracy of the assemblies.

The assembled mitogenome of *A. takesimana* is 291,633 bp in length with 45.3% GC content (GenBank accession no. PZ835188; Figure 1A) and was assembled with an average sequencing depth of 298.9× (Figure 1B). The mitogenome contains a total of 58 annotated genes, including 34 protein-coding genes, 3 ribosomal RNA (rRNA) genes, and 21 transfer RNA (tRNA) genes. The protein-coding genes comprise nine NADH dehydrogenase subunit genes (*nad1*, *nad2*, *nad3*, *nad4*, *nad4L*, *nad5*, *nad6*, *nad7*, and *nad9*), one succinate dehydrogenase gene (*sdh4*), one cytochrome b gene (*cob*), three cytochrome c oxidase genes (*cox1*, *cox2*, and *cox3*), five ATP synthase genes (*atp1*, *atp4*, *atp6*, *atp8*, and *atp9*), four cytochrome c biogenesis genes (*ccmB*, *ccmC*, *ccmFc*, and *ccmFn*), three large ribosomal protein genes (*rpl2*, *rpl5*, and *rpl16*), and six small ribosomal protein genes (*rps3*, *rps4*, *rps7*, *rps12*, *rps14* and *rps19*). In addition, the genome encodes one maturase gene (*matR*), one membrane transport protein gene (*mttB*), three rRNA genes (*rrn5*, *rrn18*, and *rrn26*), and 21 tRNA genes which includes; *trn*S-UGA, *trn*fM-CAU, *trn*M-CAU, *trn*Q-UUG, *trn*S-GGA, *trn*F- GAA, *trn*P-UGG, *trn*D-GUC (X2), *trn*C-GCA, *trn*N-GUU, *trn*Y-GUA (X3), *trn*H-GUG, *trn*E-UUC, *trn*I-CAU, *trn*G-GCC, *trn*W-CCA, *trn*S-GCU, *trn*K-UUU, *trn*S-CGA, *trn*T-GGU and *trn*L-CAA (Table 1).

Among the identified tRNA genes, *trnL-CAA*, *trnS-GGA*, and *trnN-G*UU were identified as being of plastid origin. In addition, the mitochondrial *rrn18* gene contains an 859 bp plastid-derived segment, accounting for approximately 47.1% of its total length (1824 bp), indicating the incorporation of plastid DNA into the mitogenome (Table 2). Comparative analysis showed that *A. takesimana* contains four MIPT regions corresponding to *trnL-CAA*, partial *rrn18*, *trnS-GGA*, and *trnN-GUU*. Homologous plastid-derived regions involving these sequences were also identified in the mitogenomes of *A. alpina* and *A. hirsuta*, providing additional comparative support for the occurrence of these MIPT regions within *Arabis* mitogenomes. Only those MIPT regions associated with plastid-derived gene insertions are shown in Table 2, while Table 3 provides the total MIPT regions identified in the mitochondrial genome, expressed in kilobases (kb).

### 3.2. Comparative Analysis of Mitogenome Gene Content

**Figure 2:**
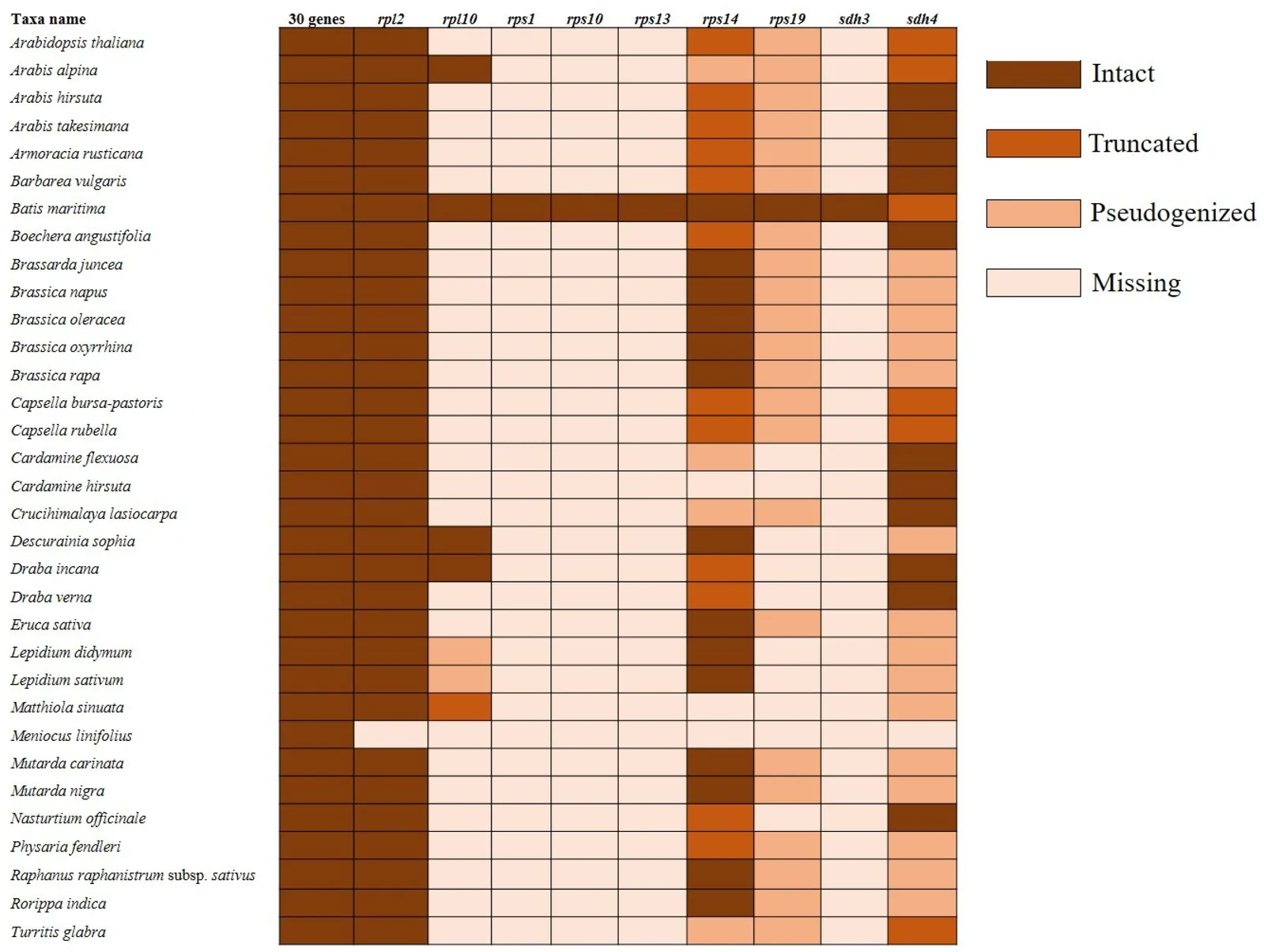
Heatmap showing the conservation status of mitochondrial CDS across 32 Brassicaceae species and the outgroup *Batis maritima*. The first column (“30 genes”) represents the conserved core set of 30 intact mitochondrial CDS present in all analyzed species. The remaining columns indicate genes exhibiting lineage-specific variation (*rpl2*, *rpl10*, *rps1*, *rps10*, *rps13*, *rps14*, *rps19*, *sdh3*, and *sdh4*). Gene status is classified as intact, truncated, pseudogenized, or missing, based on genome annotation and sequence integrity.

Comparative analysis of protein-coding genes across 32 Brassicaceae species and the outgroup, *Batis maritima*, revealed that 30 protein-coding genes (CDSs) were conserved and intact in all species. In contrast, several ribosomal protein genes and the *sdh4* gene exhibited lineage-specific gene loss, pseudogenization, or truncation (Figure 2 and Figure S1A–D). The large ribosomal protein gene *rpl2* was absent in *Meniocus linifolius* (Stephan ex Willd.) DC., whereas it was intact in all other analyzed species. The *rpl10* was present as an intact copy in *Arabis alpina*, *Descurainia Sophia* (L.) Webb. ex Prantl, *Draba incana* L., and *Batis maritima*. In contrast, it was truncated in *Matthiola sinuata* (L.) W.T. Aiton and pseudogenized in *Lepidium didymium* L. and *Lepidium sativum* L. Among the small ribosomal protein genes, *rps1*, *rps10*, and *rps13* were detected only in the outgroup species, *Batis maritima*, and were absent from all examined Brassicaceae species. The *rps14* gene was retained as an intact copy in *Brassarda juncea* (L.) Su Liu & Z.H. Feng, all four *Brassica* L. species, *Descurainia sophia*, *Eruca sativa* Mill., *Lepidium didymum*, *L. sativum*, both *Mutarda* Bernh. species, *Raphanus raphanistrum* subsp. *sativus*, *Rorippa indica* (L.) Schmalh., and *Batis maritima*. In contrast, an intact copy of *rps19* was identified only in *Batis maritima*. In Brassicaceae, *rps19* was pseudogenized in two *Cardamine* L. species, two *Draba* Dill. ex L. species, *Descurainia sophia*, both *Lepidium* species, *Matthiola sinuata*, *Meniocus linifolius*, and *Nasturtium officinale* W.T. Aiton, while it was completely absent from the remaining species. The *sdh4* gene, which encodes a subunit of mitochondrial succinate dehydrogenase (SDH), was maintained as an intact copy in *Arabis hirsuta*, *A. takesimana*, *Armoracia rusticana* G. Gaertn., B. Mey. & Scherb., *Barbarea vulgaris* W.T. Aiton, *Boechera angustifolia* (Nutt.) Dorn, both *Cardamine* species, *Crucihimalaya lasiocarpa* (Hook.f. & Thomson) Al-Shehbaz, O’Kane & R.A. Price, both *Draba* species, and *Nasturtium officinale*. A truncated copy was observed in *Arabidopsis thaliana*, *Arabis alpina*, *Batis maritima*, both *Capsella* Medik. species, and *Turritis glabra* L. The gene was completely absent only in *Meniocus linifolius*, whereas in all remaining species it was present as a pseudogene.

### 3.3. Structural Composition and Genome Size Variation in Sampled Brassicaceae Mitogenomes

**Figure 3:**
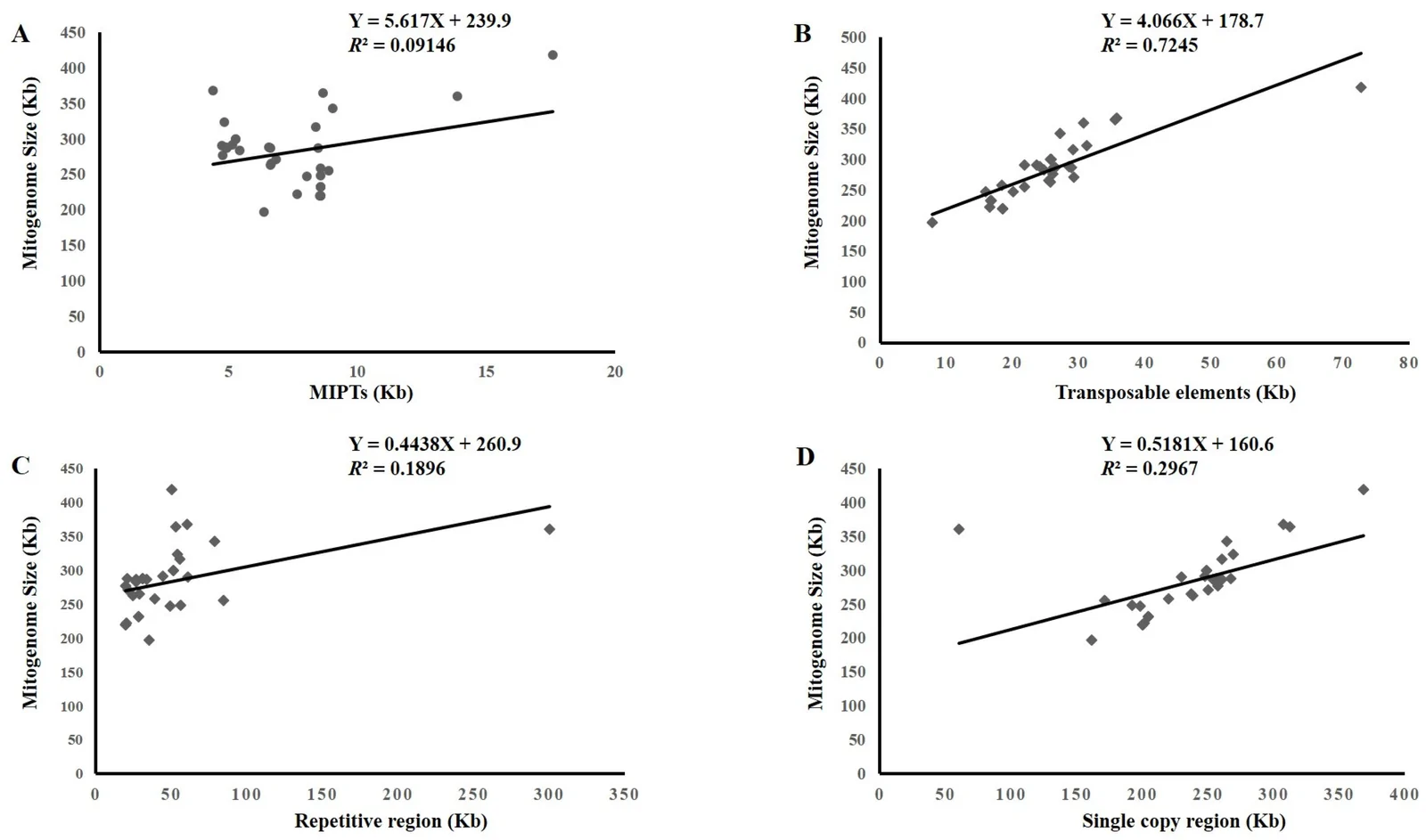
Scatter plots showing the relationships between mitogenome size and major genomic traits across 32 Brassicaceae species. (**A**) Dependence (regression) between mitogenome size and the total length of MIPTs. (**B**) Dependence (regression) between mitogenome size and TE content. (**C**) Dependence (regression) between mitogenome size and repetitive DNA content. (**D**) Dependence (regression) between mitogenome size and single-copy region length. Each point represents one species, and the solid line indicates the linear regression fit. The regression equation and coefficient of determination (*R*^2^) are shown for each regression, demonstrating a strong positive dependence of mitogenome size on transposable element (TE) content (*R*^2^ = 0.7245), whereas MIPT content (*R*^2^ = 0.09146), repetitive DNA content (*R*^2^ = 0.1896), and single-copy region length (*R*^2^ = 0.2967) show weak to moderate explanatory relationships with mitogenome size.

Gene content, TEs, MIPTs, and repetitive regions were characterized across the 32 Brassicaceae mitogenomes analyzed (Table 3). All mitogenomes contained three ribosomal RNA genes (*rrn5*, *rrn18*, and *rrn26*). The number of tRNA genes ranged from 14 to 23, whereas the number of intact CDSs varied from 30 to 33. Mitogenome size exhibited substantial variation across the family, ranging from 196,604 bp in *Meniocus linifolius* to 418,526 bp in *Matthiola sinuata*, representing a 2.12-fold difference. MIPT content accounted for 1.20–4.20% of the mitogenomes, corresponding to 4.41–17.59 kb of plastid-derived DNA. Regression analysis revealed a very weak positive association between mitogenome size and MIPT content (*R*^2^ = 0.09146; Figure 3A), suggesting a limited relationship between MIPT content and mitogenome size variation. TEs comprised 3.96–17.35% of the mitogenomes, with total TE content ranging from 7.79 to 72.64 kb. In contrast, a strong positive association was observed between mitogenome size and TE content (*R*^2^ = 0.7245; Figure 3B), suggesting that TE accumulation may be an important factor associated with mitogenome size variation. Repetitive regions represented 7.06–83.34% of the mitogenomes. Notably, in *Brassica oleracea* L., repetitive sequences occupied 83.34% of the mitogenome, greatly exceeding the single-copy fraction (16.66%). Despite the extensive variation in repetitive DNA content, regression analysis revealed only a weak positive dependence of mitogenome size on repetitive region size (*R*^2^ = 0.1896; Figure 3C), with repetitive regions explaining 18.96% of the variation in mitogenome size. Similarly, the size of single-copy regions showed a weak positive dependence on mitogenome size (*R*^2^ = 0.2967; Figure 3D), explaining 29.67% of the observed variation. Among the three *Arabis* species examined, *A. takesimana* possessed the smallest mitogenome, whereas *A. hirsuta* had the largest. TE content was relatively conserved, ranging from 7.48% to 9.65% of the mitogenome. Likewise, MIPT content was comparable between *A. alpina* (1.50%) and *A. takesimana* (1.76%). The repetitive region was smallest in *A. takesimana* (44.03 kb; 15.09% of the mitogenome) and largest in *A. hirsuta* (78.46 kb; 22.88% of the mitogenome).

### 3.4. Sequence Diversity and Selective Pressure on Conserved Mitochondrial Genes

**Figure 4:**
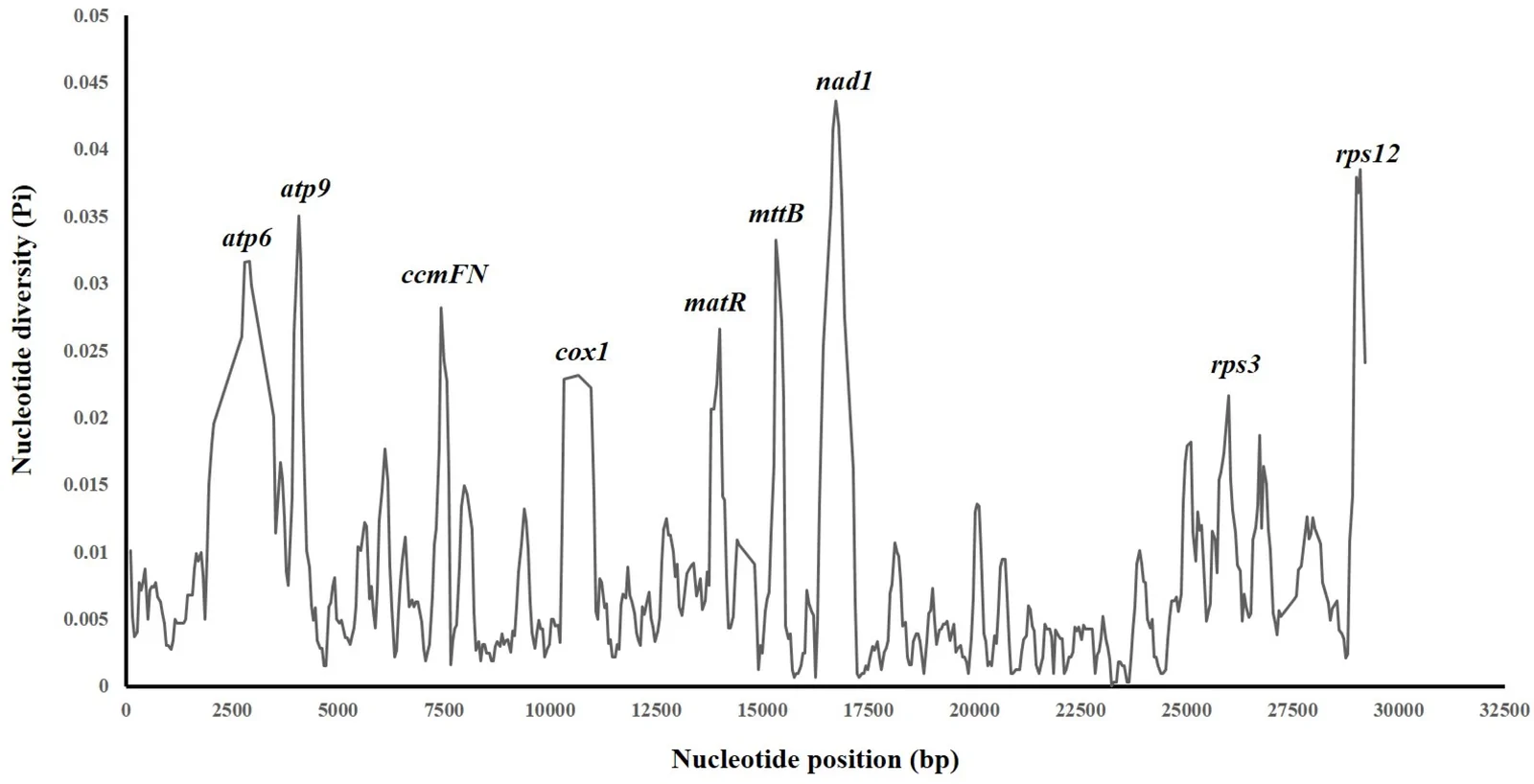
Sliding-window analysis of nucleotide diversity (*Pi*) across the concatenated alignment of 30 conserved mitochondrial CDSs from 32 Brassicaceae species. The *x*-axis represents nucleotide positions in the concatenated coding sequence alignment, and the *y*-axis indicates nucleotide diversity (*Pi*). Peaks correspond to regions of elevated sequence variation.

**Figure 5:**
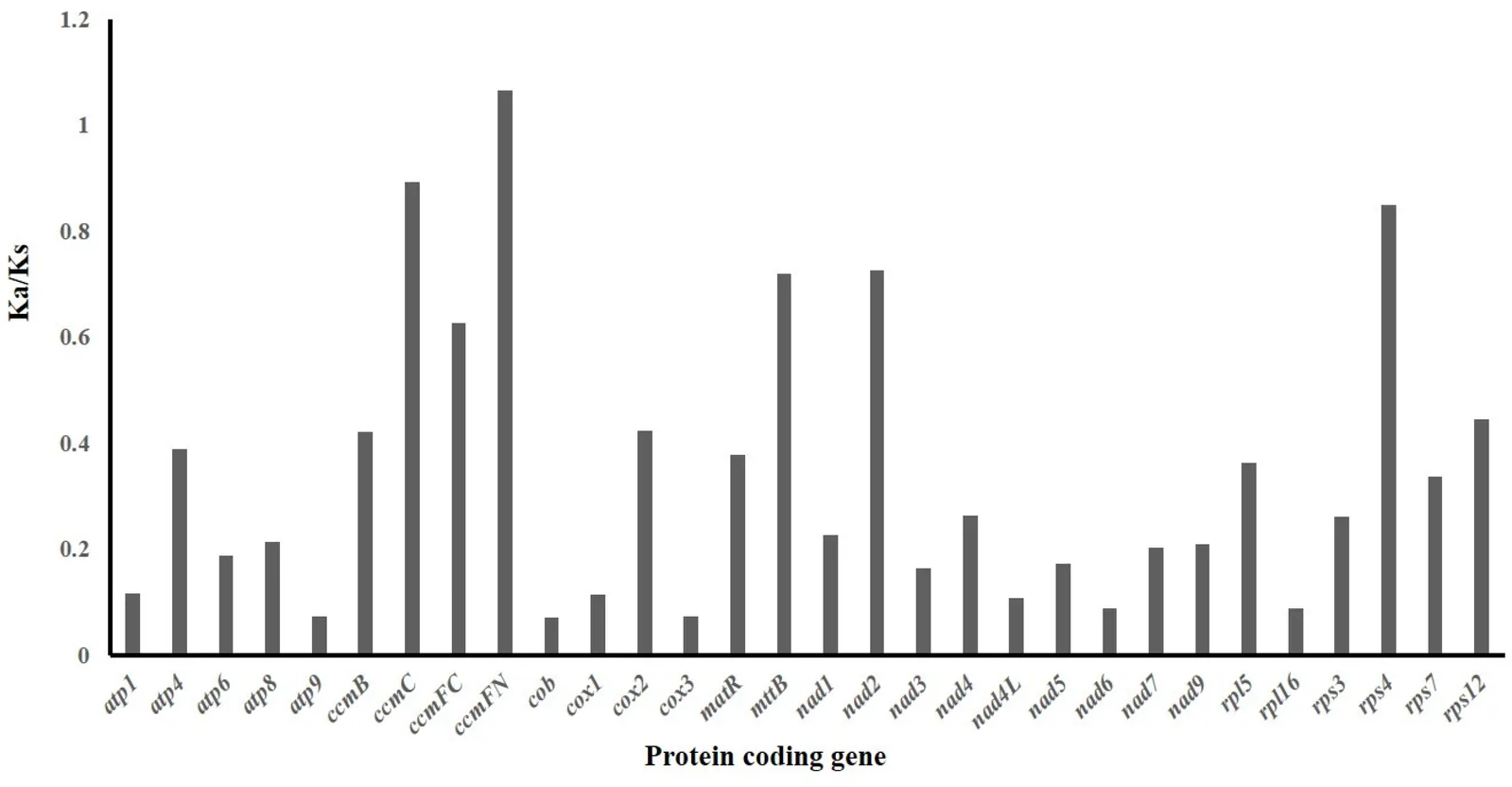
Bar plot showing the nonsynonymous-to-synonymous substitution rate ratio (Ka/Ks) for the 30 conserved mitochondrial CDSs across 32 Brassicaceae species. The x-axis represents individual protein-coding genes, and the y-axis indicates the Ka/Ks ratio.

Nucleotide diversity (*Pi*) analysis of the 30 conserved mitochondrial CDSs across 32 Brassicaceae mitogenomes revealed *Pi* values ranging from 0 to 0.04363, with a mean of 0.00797. Nine genes were identified as nucleotide diversity hotspots, displaying relatively high *Pi* values: *nad1* (0.04363), *rps12* (0.03792), *atp9* (0.03505), *mttB* (0.03324), *atp6* (0.03163), *ccmFN* (0.02815), *matR* (0.02663), *cox1* (0.02314), and *rps3* (0.02159) (Figure 4). To assess the evolutionary rates of mitochondrial CDSs, the nonsynonymous-to-synonymous substitution rate ratio (Ka/Ks) was calculated for each conserved CDS across the Brassicaceae mitogenomes. The Ka/Ks values ranged from 0.07 for *cob* to 1.065 for *ccmFN* (Figure 5).

### 3.5. Phylogenetic Analysis

**Figure 6:**
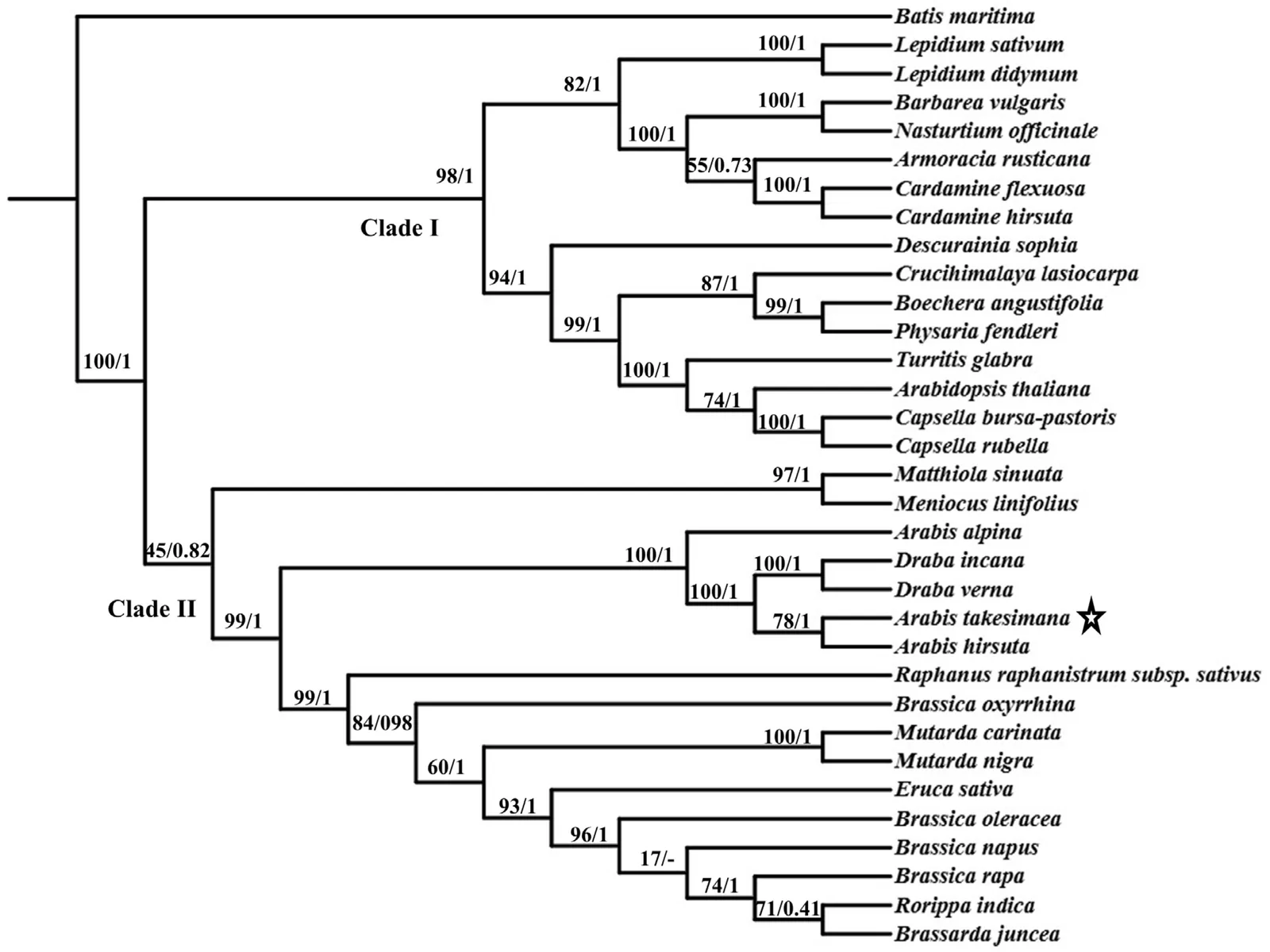
Cladogram inferred from the concatenated nucleotide sequences of 30 conserved mitochondrial CDSs from 32 Brassicaceae species, with *Batis maritima* used as the outgroup. Numbers at the nodes indicate maximum likelihood bootstrap support (BS) and Bayesian posterior probability (PP), respectively. The phylogenetic position of *A. takesimana* is indicated by a star (★).

ML and BI phylogenetic analyses based on 30 conserved CDSs from 32 sampled Brassicaceae species, with *Batis maritima* as the outgroup, resolved two major clades (Figure 6). Clade I received strong statistical support (ML BS = 98%; BI PP = 1.00) and comprised species of *Lepidium*, *Barbarea vulgaris*, *Nasturtium officinale*, *Armoracia rusticana*, two *Cardamine* species, *Crucihimalaya lasiocarpa*, *Boechera angustifolia*, *Physaria fendleri* (A. Gray) O’Kane & Al-Shehbaz, *Turritis glabra*, *Arabidopsis thaliana*, and two *Capsella* species. Clade II was moderately supported (ML BS = 45%; BI PP = 0.82) and included our studied species *A. takesimana*, which formed a well-supported sister relationship with *A. hirsuta* (ML BS = 78%; BI PP = 1.00). This clade also contained *Matthiola sinuata*, *Meniocus linifolius*, *Arabis alpina*, two *Draba* species, *Raphanus raphanistrum* subsp. *sativus*, four *Brassica* species, two *Mutarda* species, *Eruca sativa*, *Rorippa indica* (L.) Hiern, and *Brassarda juncea.*

## 4. Discussion

To date, numerous studies have reported mitogenomes from members of the Brassicaceae and performed comparative genomic analyses with related taxa [10,12,15,30]. However, most of these studies have focused primarily on phylogenetic relationships, while comprehensive comparative analyses of mitogenome structural variation across the family remain limited. In this study, we assembled and characterized the mitochondrial genome of the Korean endemic species *A. takesimana*. We further performed a comprehensive comparative analysis of its mitogenome with those of other *Arabis* species and representative members of the Brassicaceae, focusing on gene content, MIPTs, TEs, and repetitive sequences, all of which may contribute to the extensive variation in mitogenome size. In addition, we conducted nucleotide diversity, selective pressure (Ka/Ks), and phylogenetic analyses. This study provides a comparative framework for investigating mitochondrial genome evolution and diversification across the sampled taxa. The generated genomic resources will support future research on plant evolution, phylogenetics, and comparative genomics.

### 4.1. Reliability of the A. takesimana Mitochondrial Genome Assembly

In the present study, the mitochondrial genome of *Arabis takesimana* was assembled from 151 bp paired-end Illumina WGS reads generated by Tamboli et al. [31]. The original dataset (~6 GB; 42,327,400 paired-end reads) contained sequences derived from nuclear, plastid, and mitochondrial genomes. In Tamboli et al. [31] authors assembled the plastome and then subsequently in present study we computationally removed plastome-related reads by mapping the WGS reads against the corresponding plastome. The remaining reads were then used for mitochondrial genome assembly. Thus, although mitochondrial DNA was not separately extracted and sequenced, sufficient mitochondrial-derived reads present within the WGS dataset enabled assembly of the mitochondrial genome. This approach has previously been successfully applied in assembling plant mitochondrial genomes. For example, Choi et al. [51] assembled mitochondrial genomes of Fabaceae species using raw Illumina reads generated originally for plastome studies [52] after computationally filtering out plastome-derived reads. Similarly, Choi et al. [53] used previously generated Illumina WGS data originally for plastome studies [52,54] to assemble mitochondrial genomes of Fabaceae species after computational removal of plastome-related reads. Comparable short-read approaches have also been successfully used for mitochondrial genome assembly in Brassicaceae, including *Arabis alpina* [9], *Brassica oleracea* [6], and *Brassica rapa* [11]. Moreover, Ni et al. [55] reported that 90 of 416 reviewed plant mitochondrial genome studies used *de novo* assembly of short-read data. The reliability of the assembly was assessed using computational evidence, including the presence of core mitochondrial protein-coding genes and read-mapping coverage. The assembled mitochondrial genome showed an average sequencing depth of 298.9× (Figure 1B), providing substantial sequence-depth support for the assembled sequence. Plant mitochondrial genomes are highly dynamic and often contain extensive repetitive sequences, making their complete resolution challenging using short-read sequencing alone. In particular, whole-genome sequencing may contain nuclear mitochondrial DNA segments (NUMTs), which can potentially complicate mitochondrial genome assembly. Therefore, short-read data cannot completely exclude the possibility of misassembly involving highly repetitive or homologous regions. In the present study, we attempted to minimize this possibility by retrieving and retaining as many mitochondrial-derived reads as possible while excluding reads showing similarity to the plastome and other non-mitochondrial sequences. The resulting mitochondrial assembly was further supported by high read coverage, continuity of the assembled sequence, and the recovery of conserved mitochondrial protein-coding genes. Comparative analysis showed that *A. takesimana* contains four gene-containing MIPTs corresponding to *trnL-*CAA, partial *rrn18*, *trnS-*GGA, and *trnN-*GUU. Homologous plastid-derived regions involving these sequences were also identified in the mitogenomes of *A. alpina* and *A. hirsuta*, providing additional comparative support for the occurrence of these MIPT regions within *Arabis* mitogenomes. Furthermore, the overall gene content, MIPT regions, repetitive sequence composition, and transposable elements of *A. takesimana* showed patterns comparable to those reported for related *Arabis* and Brassicaceae mitogenomes. Notably, these genomic characteristics were also consistent with those of *A. hirsuta*, whose mitogenome was assembled using long-read sequencing [13]. Together, these independent comparative features provide additional support for the biological plausibility of the *A. takesimana* assembly. Nevertheless, because a complete nuclear genome of *A. takesimana* is currently unavailable, the possible contribution of NUMTs cannot be completely assessed. Future validation using long-read and/or mitochondrial DNA-enriched sequencing would therefore be valuable for resolving complex repetitive regions and further evaluating potential NUMTs.

### 4.2. Comparative Mitogenome Traits of Arabis Species

To date, mitogenome have been reported for only two *Arabis* species, *A. alpina* and *A. hirsuta* [9,13]. In the present study, we expand the genomic resources available for the genus by reporting the mitogenome of *A. takesimana*. The mitogenome of *A. takesimana* is smaller than those of the other two *Arabis* species. Despite this difference in genome size, all three species possess the same number of intact CDSs and rRNA genes, whereas the number of tRNA genes ranges from 21 to 23. The abundance of TEs is relatively conserved among the three species, accounting for 7.48–9.65% of the mitogenome. Similarly, MIPT content is comparable between *A. alpina* (1.50%) and *A. takesimana* (1.76%). In contrast, the amount of repetitive DNA varies substantially, with *A. takesimana* containing the smallest repetitive fraction (44.03 kb; 15.09% of the mitogenome) and *A. hirsuta* the largest (78.46 kb; 22.88% of the mitogenome). These findings suggest that differences in repetitive DNA, rather than gene content or TE and MIPT abundance, are a major contributor to mitogenome size variation within the genus *Arabis*.

### 4.3. Factors Influencing Mitogenome Size Variation in Sampled Brassicaceae

Mitogenome sizes within the sampled Brassicaceae species exhibit relatively modest variation, ranging from 196,604 bp to 418,526 bp, representing an approximately 2.12-fold difference. In contrast, mitogenome sizes across angiosperms display extraordinary diversity, spanning from approximately 66 kb in *Viscum scurruloideum* Barlow [56] to 11.3 Mb in *Silene conica* L. [57], corresponding to an approximately 171-fold difference. The comparatively narrow range of mitogenome sizes observed within the Brassicaceae suggests that mitogenome size has remained relatively conserved during the evolutionary history of the family, despite the extensive size variation documented across angiosperms. Such variation is widely attributed to multiple factors, including the expansion of repetitive sequences, the incorporation of foreign DNA particularly plastid-derived sequences and the proliferation of transposable elements [58,59,60].

Among the genomic traits examined, TE content showed the strongest positive association with mitogenome size in the sampled Brassicaceae taxa (*R*^2^ = 0.7245). TEs accounted for 3.96–17.35% of the mitogenomes analyzed. These findings suggest that TE accumulation may contribute to mitogenome size variation; however, this association should be interpreted cautiously because phylogenetic non-independence was not considered. The proportion of MIPTs ranged from 1.20% to 4.20% across the species examined. A weak association was observed between MIPT content and mitogenome size (*R*^2^ = 0.09146), suggesting a limited relationship between MIPT content and mitogenome size variation. This observation is consistent with previous findings in Fabaceae [51], where MIPTs likewise represented a small proportion of mitogenomes and showed a weak association with mitogenome size. However, these relationships should be interpreted cautiously because phylogenetic non-independence was not considered.

Repetitive DNA showed a weak association with mitogenome size (*R*^2^ = 0.1896), suggesting a limited relationship between repeat content and mitogenome size variation across the Brassicaceae. However, this association should be interpreted cautiously because phylogenetic non-independence was not considered. Nevertheless, notable exceptions were observed. In *Brassica oleracea*, repetitive sequences accounted for 83.34% of the mitogenome, far exceeding the single-copy fraction (16.66%). This unusually high repeat content results from the presence of two large dispersed repeats, each spanning approximately 144.5 kb. The first repeat extends from positions 3294 to 147,844 bp, while the second spans positions 173,327 to 317,876 bp. Consistent with this observation, Chang et al. [7] reported that the comparatively large mitogenome of *B. oleracea* arose from the duplication of a 141.8 kb genomic segment. Similar cases have been documented in other angiosperms, in *Vicia faba* L. repeats comprise 60.6% of the mitogenome, exceeding the proportion of single-copy sequences [51]. These observations suggest that repeat accumulation may contribute to mitogenome expansion in some lineages, but its association with mitogenome size appears to vary among taxa. This variation may reflect differences in evolutionary history, selective pressures, and stochastic genomic rearrangements.

### 4.4. Evolutionary Patterns of Mitochondrial Gene Retention and Loss in Sampled Brassicaceae

Comparative analysis of the CDSs across sampled Brassicaceae species and the outgroup species, *Batis maritima*, identified a conserved core set of 30 intact mitochondrial CDSs, including genes involved in oxidative phosphorylation (*atp*, *cox*, *cob*, and *nad*), cytochrome *c* biogenesis (*ccm*), the maturase gene (*matR*), the membrane transport gene (*mttB*), and a limited number of ribosomal protein genes (*rpl5*, *rpl16*, *rps3*, *rps4*, *rps7*, and *rps12*). This conserved gene set is consistent with the core mitochondrial gene complement reported across most vascular plants [61]. In contrast, the remaining ribosomal protein genes, particularly members of the *rps* and *rpl* gene families and succinate dehydrogenase (*sdh4*), exhibited substantial variation in coding status, occurring as intact, truncated, pseudogenized, or completely absent genes among the analyzed species.

Among the CDS examined, *rps1*, *rps10*, *rps13*, and *sdh3* were retained as intact genes exclusively in the outgroup species, *Batis maritima* (Bataceae), whereas none of the analyzed Brassicaceae species possessed functional copies of these genes. The *rps19* gene was detected only as a pseudogene in Brassicaceae, while remaining intact in *B. maritima*. This pattern is in strong agreement with the comparative analysis of Brassicales mitogenomes by Grewe et al. [6], who reported that the losses of *rps1*, *rps10*, and *rps19* are shared synapomorphic traits of Brassicaceae, whereas the losses of *rps13* and *sdh3* occurred earlier and are shared more broadly across Brassicales. Our results therefore confirm that these gene losses have been evolutionarily stable throughout the diversification of Brassicaceae and have been maintained across multiple lineages within the family. The consistent absence of intact copies of these genes in all examined Brassicaceae species suggests that their loss occurred early during the evolution of the family and predates the divergence of extant Brassicaceae lineages. The retention of intact copies in *Batis maritima* further supports the hypothesis that these genes were present in the ancestral Brassicales mitogenome and were subsequently lost in the Brassicaceae lineage. As proposed by Adams and Palmer [62], the loss of mitochondrial ribosomal protein and respiratory genes is frequently accompanied by functional transfer to the nuclear genome, where nuclear-encoded proteins are imported back into the mitochondria. Consequently, the absence of these genes from the mitogenome is unlikely to reflect a loss of function but rather an evolutionary shift in genomic location. The concordance between our findings and previous comparative studies suggests that gene loss, particularly involving ribosomal protein genes, is a recurrent feature of mitochondrial genome evolution among the sampled Brassicaceae lineages. However, given the limited taxonomic sampling, this pattern should be considered preliminary and requires further evaluation across a broader representation of the family.

### 4.5. Nucleotide Diversity, Selection Pressure, and Phylogenetic Relationships

Nucleotide diversity analysis identified nine CDS as diversity hotspots across the analyzed Brassicaceae species: *nad1*, *rps12*, *atp9*, *mttB*, *atp6*, *ccmFN*, *matR*, *cox1*, and *rps3*. In contrast, Liu et al. [15] identified *cox2*, *ccmFc*, *nad2*, and *nad4* as the most variable mitochondrial genes among *Brassica* species. The differences between our findings and those of Liu et al. [15] likely reflect the broader taxonomic sampling in the present study, which encompasses multiple genera across the Brassicaceae rather than a single genus. Consequently, the variable genes identified here represent nucleotide diversity hotspots at the family level and may serve as valuable molecular markers for phylogenetic, phylogeographic, population genetic, and species identification studies within the Brassicaceae.

The average Ka/Ks ratio across the 30 conserved mitochondrial CDS was 0.34, indicating that most genes are evolving under strong purifying selection. This pattern reflects substantial evolutionary constraints on mitochondrial coding sequences in the Brassicaceae (Figure 5). Among the analyzed genes, *ccmFN* was the only gene with a Ka/Ks ratio slightly greater than 1, suggesting that it may have experienced positive selection during the evolutionary history of the family. These findings are largely consistent with those of Liu et al. [15], who also reported an average Ka/Ks ratio of 0.34 for mitochondrial CDS in *Brassica* species. In their study, *ccmFN*, *ccmC*, *cox2***,** and *rpl2* exhibited Ka/Ks ratios greater than 1.0, indicating potential positive selection, whereas the remaining genes showed Ka/Ks ratios below 1, consistent with purifying selection. The differences in the positively selected genes between the two studies are likely attributable to the broader taxonomic sampling in the present analysis, which includes representatives from across the Brassicaceae rather than only the genus *Brassica*. In contrast, the *cob* gene exhibited the lowest Ka/Ks ratio (0.07) and the smallest level of sequence variation among all analyzed genes, indicating exceptionally strong purifying selection. This high degree of conservation suggests that *cob* is an evolutionarily constrained mitochondrial gene that plays an essential role in mitochondrial function and energy metabolism across the Brassicaceae.

Phylogenetic analyses based on the 30 conserved mitogenome CDS resolved the sampled Brassicaceae species into two major clades with strong statistical support. The relationships among *Brassica* species recovered in our phylogeny are consistent with those reported by Liu et al. [15], who also reconstructed mitochondrial phylogenies using conserved CDS. Within the genus *Arabis*, *A. takesimana* was recovered as the sister lineage to *A. hirsuta*, while *A. alpina* formed a sister lineage to the clade comprising *A. takesimana*, *A. hirsuta*, and the two *Draba* species. This phylogenetic placement is consistent with the plastome-based phylogeny reported by Tamboli et al. [31], suggesting a concordance between mitochondrial and plastid genomes in resolving phylogenetic relationships within this genus.

## 5. Conclusions

This study expands the mitogenomic resources available for the Brassicaceae by reporting the mitogenome of the Korean endemic species *A. takesimana* and assessing its phylogenetic position among the sampled Brassicaceae species based on conserved mitochondrial CDS. The mitogenome of *A. takesimana* contains an 5.15 kb MIPT region, from which three intact tRNA genes (*trnA*, *trn*L-CAA, *trn*S-GGA, and *trn*N-GUU) and a partial *rrn18* gene were derived. In addition, the mitogenome harbors 21.83 kb of transposable element-derived sequences and 44.03 kb of repetitive DNA, accounting for 7.48% and 15.09% of the total mitogenome size, respectively. In addition to characterizing the *A. takesimana* mitogenome, We conducted a comparative analysis of 32 Brassicaceae mitogenomes to examine patterns of genome evolution, gene content, structural variation, and phylogenetic relationships among the sampled taxa. Comparative gene content analysis revealed a conserved core set of 30 intact CDSs across the sampled Brassicaceae taxa, whereas members of the *rps* and *rpl* gene families and the succinate dehydrogenase gene *sdh4* exhibited substantial variation in coding status, occurring as intact, truncated, pseudogenized, or completely absent among different species. Furthermore, our analyses provide a comprehensive assessment of the contributions of MIPTs, transposable elements, and repetitive sequences to mitogenome size variation across the sampled Brassicaceae species.

## Figures and Tables

**Table 1:** Gene composition in the mitogenome of *A. takesimana.*

Group of Genes	Gene Name	Number
ATP synthase	*atp1*, *atp4*, *atp6*, *atp8*, *atp9*	5
Cytochrome c biogenesis	*ccmB*, *ccmC*, *ccmFc* *, *ccmFn* (X2)	4
Ubiquinol cytochrome c reductase	*cob*	1
Cytochrome c oxidase	*cox1*, *cox2* *, *cox3*	3
Maturases	*matR*	1
Transport membrane protein	*mttB*	1
NADH dehydrogenase	*nad1* *, *nad2* *, *nad3*, *nad4* *, *nad4L*, *nad5* *, *nad6*, *nad7* *, *nad9*	9
Large subunit of ribosomal proteins	*rpl2* *, *rpl5*, *rpl16*	3
Small subunit of ribosomal proteins	*rps3* *, *rps4*, *rps7*, *rps12*, *rps14*, *rps19*	6
Succinate dehydrogenase	*sdh4*	1
Ribosomal RNAs	*rrn5*, *rrn18*, *rrn26*	3
Transfer RNAs	*trn*S-UGA, *trn*fM-CAU, *trn*M-CAU, *trn*Q-UUG, *trn*S-GGA, *trn*F-GAA (X2), *trn*P-UGG, *trn*D-GUC (X2), *trn*C-GCA, *trn*N-GUU, *trn*Y-GUA (X3), *trn*H-GUG, *trn*E-UUC, *trn*I-CAU, *trn*G-GCC, *trn*W-CCA, *trn*S-GCU, *trn*K-UUU, *trn*S-CGA, *trn*T-GGU, *trn*L-CAA	21
Total		58

Note: * indicates genes containing one or more introns; X2, X3 indicates genes present in multiple copies in the mitogenome.

**Table 2:** Gene-containing mitochondrial plastid DNA (MIPT) insertions in *Arabis* species.

Taxa Name	Sequence	Position (bp)	Gene
*A. alpina*	MIPT-1	255483-256341	Partial *rrn18*
	MIPT-2	277552-277761	*trn*N-GUU
	MIPT-3	279664-281166	*trn*L-CAA
*A. hirusta*	MIPT-1	88555-88667	*trn*S-GGA
	MIPT-2	106092-107256	*trn*N-GUU
	MIPT-3	126342-127200	Partial *rrn18*
	MIPT-4	236248-238106	*trn*L-CAA
	MIPT-5	277985-281470	*trn*A-UGC
*A. takesimana*	MIPT-1	4903-6765	*trn*L-CAA
	MIPT-2	30359-31217	Partial *rrn*18
	MIPT-3	213989-214101	*trn*S-GGA
	MIPT-4	238336-238827	*trn*N-GUU

**Table 3:** General features of mitogenomes from 32 Brassicaceae species analyzed in this study.

Taxa	Genome Size (bp)	Number of Unique Intact Genes			Transposable Elements		MIPTs		Single Copy Region		Repetitive Region	
		Protein-Coding	rRNA	tRNA	%	kb	%	kb	%	kb	%	kb
*Arabidopsis thaliana*	367,808	31	3	20	9.70	35.71	1.20	4.41	83.61	307.51	16.39	60.29
*Arabis alpina*	323,159	32	3	21	9.65	31.20	1.50	4.85	83.36	269.39	16.64	53.76
*Arabis hirsuta*	342,942	32	3	23	7.91	27.12	2.63	9.05	77.12	264.47	22.88	78.46
*Arabis takesimana*	291,633	32	3	22	7.48	21.83	1.76	5.15	84.91	247.60	15.09	44.03
*Armoracia rusticana*	316,393	32	3	19	9.19	29.08	2.64	8.38	82.41	260.67	17.59	55.63
*Barbarea vulgaris*	364,652	32	3	18	9.72	35.45	2.37	8.67	85.57	312.04	14.43	52.60
*Boechera angustifolia*	271,601	32	3	18	10.77	29.27	2.51	6.84	92.08	250.09	7.92	21.50
*Brassarda juncea*	219,766	32	3	20	8.41	18.50	3.89	8.56	90.98	199.92	9.02	19.84
*Brassica napus*	221,853	32	3	20	7.45	16.53	3.45	7.66	90.79	201.41	9.21	20.44
*Brassica oleracea*	360,271	32	3	21	8.53	30.75	3.85	13.87	16.66	60.02	83.34	300.25
*Brassica oxyrrhina*	247,936	32	3	21	6.42	15.94	3.45	8.56	77.37	191.83	22.63	56.10
*Brassica rapa*	219,747	32	3	21	8.43	18.52	3.89	8.56	90.97	199.90	9.03	19.83
*Capsella bursa-pastoris*	287,799	31	3	18	9.89	28.48	1.70	4.91	89.22	256.75	10.78	31.04
*Capsella rubella*	287,405	31	3	18	10.02	28.81	1.71	4.92	89.23	256.43	10.77	30.96
*Cardamine flexuosa*	299,980	32	3	18	8.58	25.74	1.76	5.29	82.91	248.71	17.09	51.26
*Cardamine hirsuta*	300,090	32	3	18	8.58	25.76	1.76	5.29	82.87	248.69	17.13	51.39
*Crucihimalaya lasiocarpa*	288,122	32	3	18	8.34	24.05	2.27	6.55	92.78	267.33	7.22	20.79
*Descurainia sophia*	265,488	33	3	18	9.60	25.48	2.51	6.67	89.21	236.83	10.79	28.65
*Draba incana*	283,085	33	3	20	8.72	24.70	1.92	5.44	90.58	256.40	9.42	26.67
*Draba verna*	290,597	32	3	20	8.14	23.66	1.63	4.75	79.01	229.60	20.99	60.98
*Eruca sativa*	247,696	32	3	19	8.10	20.07	3.24	8.04	80.07	198.32	19.93	49.30
*Lepidium didymum*	262,678	32	3	19	9.80	25.75	2.53	6.64	90.62	238.03	9.38	24.64
*Lepidium sativum*	287,054	32	3	18	9.12	26.20	2.31	6.64	90.81	260.66	9.19	26.38
*Matthiola sinuata*	418,526	31	3	19	17.35	72.64	4.20	17.59	87.98	368.21	12.02	50.31
*Meniocus linifolius*	196,604	30	3	14	3.96	7.79	3.25	6.39	82.04	161.30	17.96	35.30
*Mutarda carinata*	232,241	32	3	20	7.18	16.68	3.69	8.58	87.92	204.19	12.08	28.04
*Mutarda nigra*	232,145	32	3	20	7.20	16.72	3.69	8.58	87.92	204.10	12.08	28.04
*Nasturtium officinale*	286,925	32	3	18	9.21	26.44	2.95	8.47	88.31	253.38	11.69	33.53
*Physaria fendleri*	255,307	31	3	14	8.54	21.82	3.47	8.88	66.97	170.98	33.03	84.32
*Raphanus raphanistrum subsp. sativus*	258,426	32	3	20	7.11	18.38	3.32	8.58	85.01	219.67	14.99	38.75
*Rorippa indica*	219,775	32	3	20	8.41	18.50	3.88	8.54	90.97	199.93	9.03	19.84
*Turritis glabra*	276,710	31	3	17	9.39	26.00	1.72	4.76	92.94	257.16	7.06	19.54

## Data Availability

The mitogenome assembled in this study has been deposited in NCBI GenBank under accession number PZ835188. The associated raw sequencing data have been submitted under BioProject ID PRJNA1521648, BioSample accession SAMN62807680, and the Sequence Read Archive (SRA) SRR40440025.

## Supplementary Materials

The following supporting information can be downloaded at: https://www.mdpi.com/article/10.3390/biology15191762/s1, Figure S1A–D: Multiple sequence alignments of the *rpl10*, *rps14*, *rps19*, and *sdh*4 coding sequences (CDSs); Table S1: List of Species with corresponding mitochondrial and plastid genomes used in MIPT Analysis.
