# Supplementary material for: Mitochondrial Genome of the Korean Endemic Arabis takesimana (Brassicaceae): Addition to the Genomic Resources and Evolutionary Insights
Source: Biology (Basel). 2026 Oct 2;15(19):1762. doi: 10.3390/biology15191762 (PMC13663962; doi:10.3390/biology15191762)
Supplement: Supplementary file 1 [file biology-15-01762-s001.zip › Figure S1A–D. Multiple sequence alignments of the rpl10, rps14, rps19, and sdh4 coding sequences (CDSs).pdf]

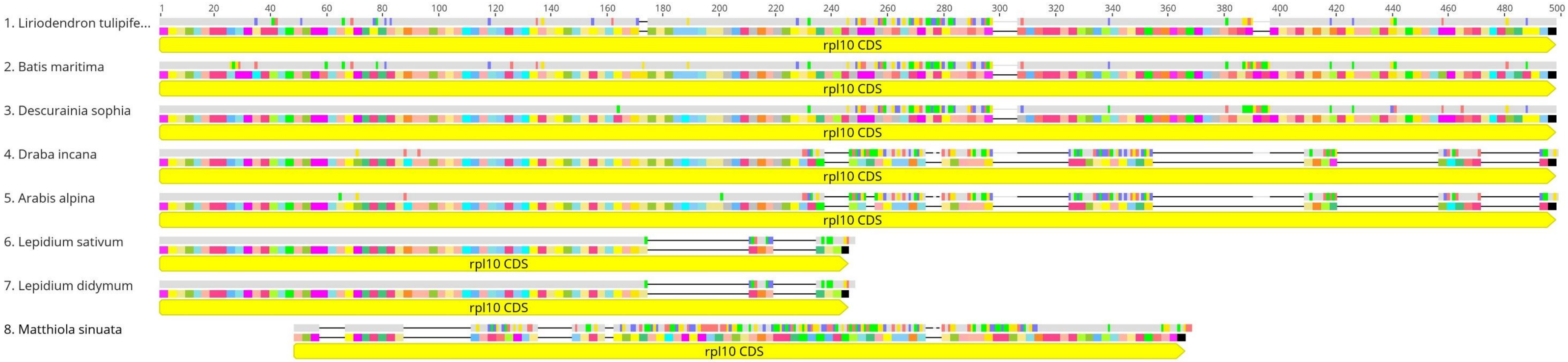

Figure S1A. Multiple sequence alignment of the mitochondrial *rpl10* coding sequence (CDS) from Brassicaceae species and the outgroup *Batis maritima*, using the intact *Liriodendron tulipifera rpl10* sequence as the reference. The alignment was used to assess the structural integrity and evolutionary status of the *rpl10* gene across the analyzed taxa.

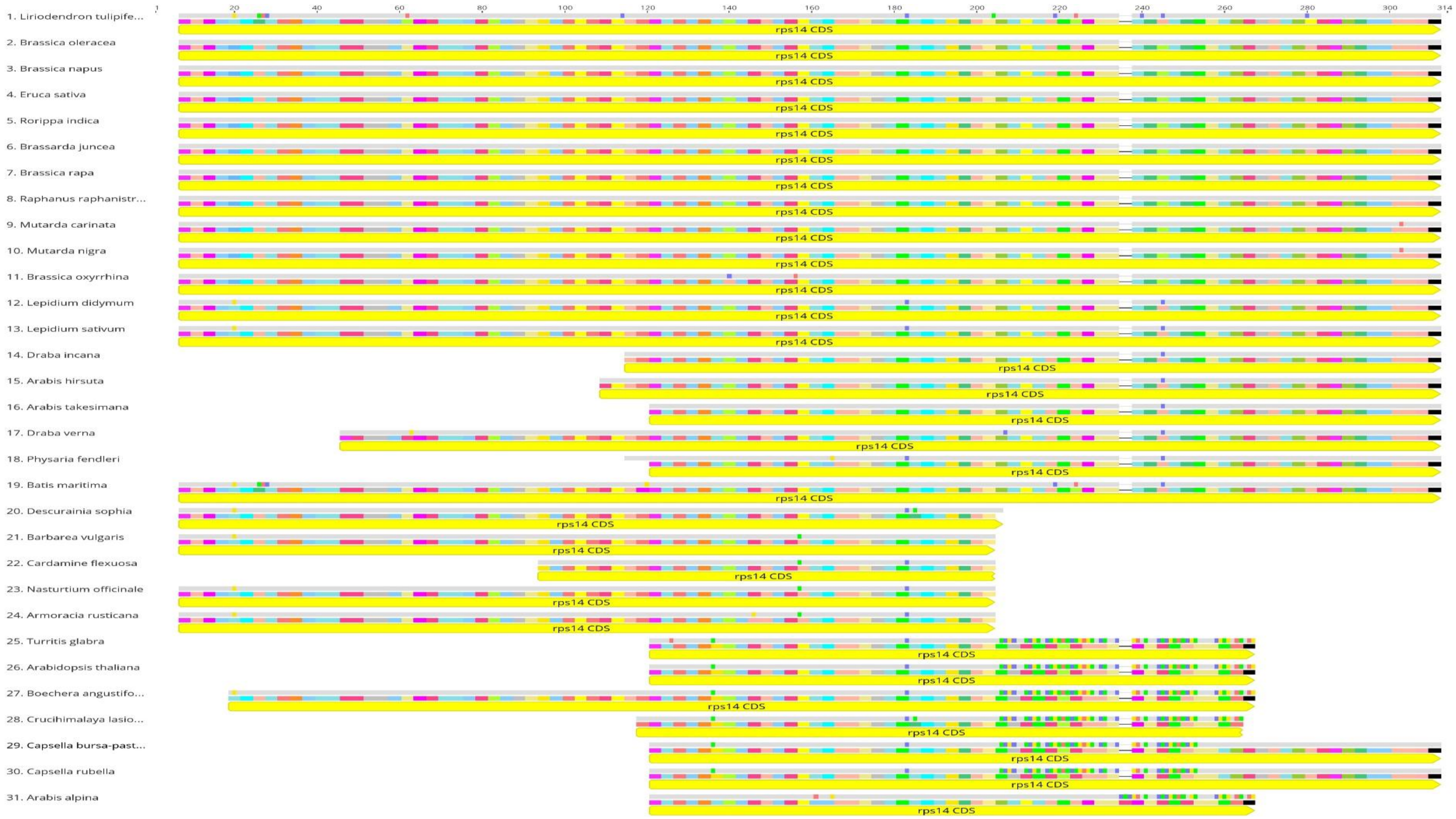

Figure S1B. Multiple sequence alignment of the mitochondrial *rps14* coding sequence (CDS) from Brassicaceae species and the outgroup *Batis maritima*, with *Liriodendron tulipifera* serving as the reference. The alignment was used to assess the structural integrity and evolutionary status of the *rps14* gene across the analyzed taxa.

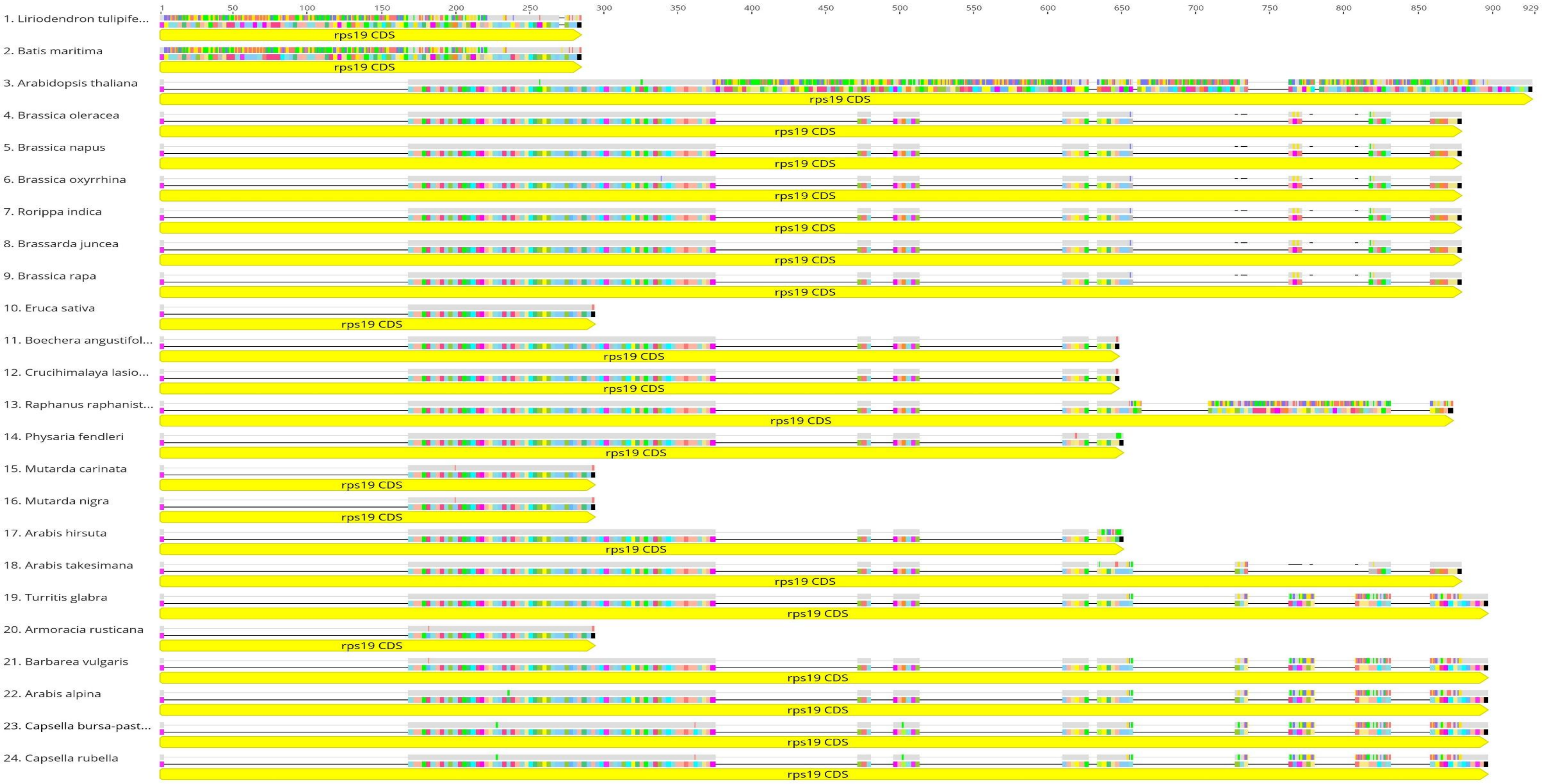

Figure S1C. Multiple sequence alignment of the mitochondrial *rps19* coding sequence (CDS) from Brassicaceae species and the outgroup *Batis maritima*, with *Liriodendron tulipifera* serving as the reference. The alignment was used to assess the structural integrity and evolutionary status of the *rps19* gene across the analyzed taxa.

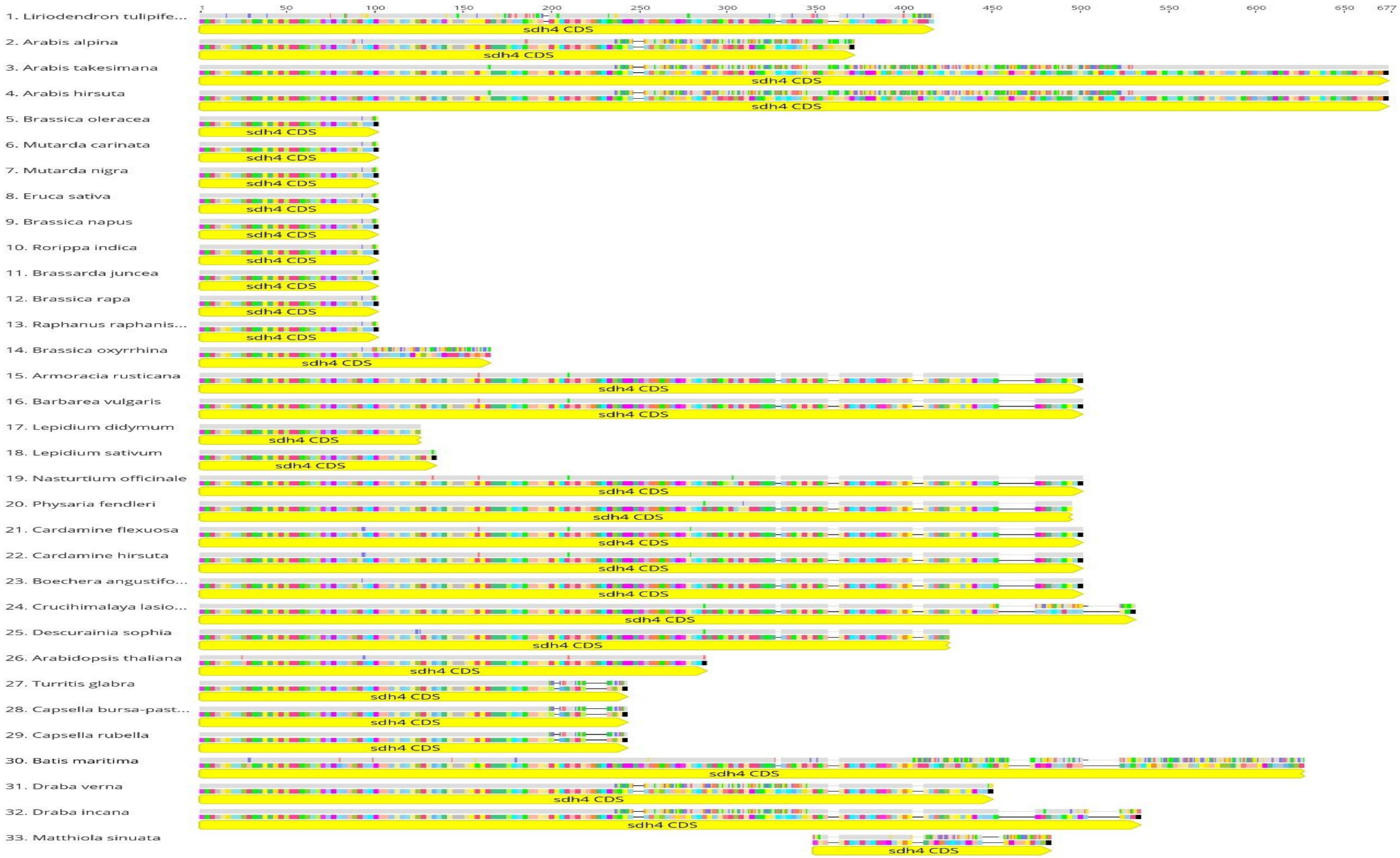

Figure S1D. Multiple sequence alignment of the mitochondrial *sdh4* coding sequence (CDS) from Brassicaceae species and the outgroup *Batis maritima*, with *Liriodendron tulipifera* serving as the reference. The alignment was used to assess the structural integrity and evolutionary status of the *sdh4* gene across the analyzed taxa
