# Supplementary material for: Mitochondrial Genome of the Korean Endemic Arabis takesimana (Brassicaceae): Addition to the Genomic Resources and Evolutionary Insights
Source: Biology (Basel). 2026 Oct 2;15(19):1762. doi: 10.3390/biology15191762 (PMC13663962; doi:10.3390/biology15191762)
Supplement: Supplementary file 1 [file biology-15-01762-s001.zip › Table S1. List of Species with corresponding mitochondrial and plastid genomes used in MIPT Analysis.pdf]

Table S1. List of Species with corresponding mitochondrial and plastid genomes used in MIPT Analysis

Species name	Accession number of mitochondrial genome	Accession number of plastome
Arabidopsis thaliana	NC_037304	AP000423
Arabis alpina	NC_037070	NC_023367
Arabis hirsuta	OZ053314	NC_009268
Arabis takesimana	PZ835188	OR813051
Armoracia rusticana	OZ278782	NC_060501
Barbarea vulgaris	OY763895	NC_081109
Boechera angustifolia	NC_042143	NC_049600 (*Boechera suffrutescens*)
Brassarda juncea	NC_016123	NC_028272
Brassica napus	AP006444	NC_016734
Brassica oleracea	NC_016118	MN396847
Brassica oxyrrhina	AP018041	NC_016734 (*Brassica napus*)
Brassica rapa	NC_016125	MT726210
Capsella bursa-pastoris	MN746809	NC_009270
Capsella rubella	NC_042883	NC_027693
Cardamine flexuosa	OY743222	OY743223
Cardamine hirsuta	OZ156426	NC_049607
Crucihimalaya lasiocarpa	NC_085700	NC_049612
Descurainia sophia	OZ254941	NC_049631
Draba incana	OY755218	NC_049627
Draba verna	OZ174244	NC_049633
Eruca sativa	KF442616	MZ855504
Lepidium didymum	OY986989	OY986990
Lepidium sativum	NC_085699	NC_047178

Matthiola sinuata	OZ209988	OZ209989
Meniocus linifolius	NC_085616	NC_049581
Mutarda carinata	NC_016120	NC_059807
Mutarda nigra	AP012989	NC_030450
Nasturtium officinale	OY978515	NC_009275
Physaria fendleri	OQ818196	BK063463
Raphanus raphanistrum subsp. sativus	NC_018551	NC_024469 (*Raphanus sativus*)
Rorippa indica	PP780168	NC_065833
Turritis glabra	LC325489	NC_061285
Outgroup		
Batis maritima	NC_024429.	-
